# Magnitude of Khat use and associated factors among women attending antenatal care in Gedeo zone health centers, southern Ethiopia: a facility based cross sectional study

**DOI:** 10.1186/s12889-019-8026-0

**Published:** 2020-01-28

**Authors:** Birhanie Mekuriaw, Zelalem Belayneh, Yimenu Yitayih

**Affiliations:** 1 Department of Psychiatry, College of Health and Medical Science, Dilla University, Dilla, Ethiopia; 2 Department of Psychiatry, Faculty of Medicine, Institute of Health, Jimma University, Jimma, Ethiopia

**Keywords:** Khat chewing, Pregnant women, Substance use, Addiction, Gedeo zone, Khat use

## Abstract

**Background:**

Khat (*Catha edulis*) is a fresh green leave commonly chewed for recreational purpose and its euphoric effect as a result of dopamine activities stimulation effect of Khat. Women may use Khat during their pregnancy to tolerate their pregnancy related distress. Khat has biological, social or psychological complications; adverse pregnancy outcomes and negatively affects the health of the mother and the fetus. However, magnitude, pattern and associated factors of Khat use are not well addressed among pregnant women in Ethiopia. The aim of this study was to address this gap by assessing the magnitude and associated factors of Khat use among women attending antenatal care service at Gedeo zone rural health centers.

**Methods:**

This was a facility based cross-sectional study conducted at rural health centers in Gedeo zone from June 1^st^ to August 1^st^, 2017. The data were collected through structured interview using an interview guide developed from different literatures. A total of 718 pregnant women attending antenatal care service participated in the study using multi stage sampling technique. Bivariable and multivariable analysis were used to identify associated factors of Khat use among women during their current pregnancy period. In the multivariable analysis, variables with *P*-Values of less than 0.05 were considered as statistically significant correlates of Khat use. The strength of the association was also measured with adjusted odds ratio at a corresponding 95% confidence interval.

**Results:**

The lifetime and current prevalence of khat use among pregnant women were 11.0% (95%CI: 8.8–13.2) and 9.9% (95% CI: 7.7–12), respectively. The odds of being khat user was higher among those who had khat user partner [AOR = 3.450, 95% C.I (1.907–6.244)], respondents with alcohol use behavior [AOR = 3.235, 95% C.I (1.573–6.659)] and mental distress [AOR = 3.575, 95% C.I (2.067–6.189)].

**Conclusion:**

Significant proportions of pregnant women were experiencing khat chewing during pregnancy. Having khat user partner, alcohol use behavior and metal distress were significantly associated with khat use of pregnant women. This demonstrates a need to integrate the prevention, early identification and intervention of Khat use as a component of treatment modality for pregnant women during their antenatal visit.

## Background

Khat (*Catha edulis*) is a fresh green leave plant commonly cultivated in eastern Africa and the Arabian Peninsula starting from several centuries [1, 2]. People commonly chew the green leaves of Khat for recreational purpose and its euphoric effect as a result of its ability to stimulate the dopamine activities of the brain [2, 3]. Now days, khat chewing practice is introduced to other parts of the world as a result of migration, easily accessibility of road networks and fast air transportation. Khat cultivation and distribution of its green leaves is considered as a great business, and chewing becomes more a common habit worldwide [3, 4].

It is estimated that about 10 million people worldwide chew khat daily to enjoy with its psycho-stimulant and euphorigenic effects [5]. Khat has been known to have psycho-active ingredient (cathinone) which has analogues structure, mechanisms of action and effects on the central nervous system with amphetamine [6]. Thus, khat chewing can cause more addiction, and people are facing difficulties to stop chewing as once they have started it. Furthermore, the dopamine activity stimulation effect of Khat has a brain rewarding system that might reinforce individuals for further chewing and combination of other psycho-active substances after their first initiation for recreational purpose or other reasons [6, 7].

Khat chewing is commonly not a solitary practice, rather people chew khat together with friends collected together and discuss freely regarding any topic considered important by the chewers themselves [8, 9]. As a result, people often attend this ceremony mainly for their socialization purpose and so as to escape from any stressful life events while not much attracted by the leaves of Khat [10]. Similarly, women may use khat chewing as a reason for establishing close relationships with their spouse and peers while they become pregnant [11]. Although Khat has short term stimulant and euphoric effects, it has linked to long term consequences which can produce biological, social or psychological impact on the mother and fetus [12, 13] and it is highly recommended to be totally avoided during pregnancy [14–16]. Khat chewing also results in adverse pregnancy outcomes such as sexual difficulties, lowering libidos, decreasing food intake, decreasing of utro-placental blood flow which may result in teratogenic effects, impairment of fetal growth and low birth weight [12, 17–19].

Despite of these diverse and devastating consequences of Khat use during pregnancy period, the focus of most studies was to assess the magnitude of Khat use among the general population and college students, but the magnitude of khat use among pregnant women and its determinant factors are not well addressed in Ethiopia. As per the authors’ knowledge, there is no any empirical evidence reporting about the magnitude, pattern and associated factors of Khat use during pregnancy in the study area. Therefore, this study aimed to fill this gap by assessing the magnitude, pattern and associated factors of Khat use among women attending Anti Natal Care. This can provide a great opportunity for professionals to consider preventive and interventional measures that can reduce pregnancy related problems which might be caused by Khat use during pregnancy. The findings of this study can also help for planners, decision makers, and clinicians by showing the magnitude, pattern and reasons for the initiation of Khat chewing during pregnancy. Furthermore, the findings of this study will be used as baseline information for further studies.

## Methods

### Study design, period and set-up

This was an institutional based study conducted from June 1^st^ to August 1^st^, 2017. The study was conducted among pregnant women of Gedeo zone attending ANC service at rural health centers. Gedeo is one of the 13 zones found in Southern Nation Nationalities and Peoples of Region (SNNPR), Southern Ethiopia. Dilla is the zonal town of Gedeo zone located at 359 km South East far from the capital city of Ethiopia (Addis Ababa). The zone has administrational classification of six districts and two administrations. Within Gedeo zone, there are about 38 health centers providing service for about 41,733 pregnant women expected to initiate the ANC service each year.

### Sample size and sampling technique

We have used a single population proportion formula to calculate the minimum sample size required for this study. Assumptions made for the sample size calculation were 50% prevalence of Khat use, 5% margin of error and 95% confidence interval (CI). As we have used multi stage sampling technique, a design effect of 2 was also considered. Finally, the total sample size was 845 by adding a 10% non-response rate.

Two-stages sampling technique was used to recruit the required study participants. In the first stage, four districts and one city administration were randomly selected from a total of six districts and two city administrations found in Gedeo zone. In the second stage, a total of eight rural health centers were also randomly selected from the pre-selected four districts and one city administration. Then, the calculated sample size was proportionally allocated to each of the eight randomly selected health centers. Finally, systematic random sampling technique was used to address individual participants from each selected health center using a sampling interval (K). The sampling interval (K) was determined by dividing the total number of eligible women attending ANC service (age greater or equal to 18 years and without serious medical illness) during the data collection period to the proportionally allocated number of samples to each health center. To identify the first participant, we used lottery method between one and K. After addressing the first participant, K value was added to recruit the next candidate until the proposed sample size was addressed.

### Data collection instruments and operational definitions

Data were collected using an interviewer administered questionnaire/interview guide. The interview guide was developed for this study after reviewing different related literatures (Additional file 1). It was first prepared in English and translated to the commonly spoken languages in the study area (Amharic and Gedeufa). Back translation to English was also done to check its accuracy. Pretest of the Amharic and Gedeufa version of the questionnaire was conducted among 43 pregnant women attending ANC service at Chuko health center. The purpose of the pretest was to check the understandability of the questionnaire, ability of the questionnaire to address proposed objectives and appropriateness of expressions for the context of the study area. Based on the pretest results, minor modification was done on the contents of the questionnaire, and some culturally sensitive expressions were rewritten by replacing appropriate statements.

The questionnaire was organized in to six sub-sections. The first section of the questionnaire had socio-demographic variables including age in years, marital status, ethnicity, religion, residency and income level. In the second part, obstetric and gynecological related factors (parity, pregnancy status, abortion history and numbers of ANC visit) were directly recorded from the women’s chart (medical record) in a confidential manner.

The third part was the Oslo-3 Social Support Scale which was used to assess level of social support. Oslo Social Support Scale is an internationally validated tool used to measure the level of social support both in clinical settings and community studies. It has three likert scale questions with five possible response options for the two questions and four options for one question [20]. The total sum score of Oslo-3 Social Support Scale ranges from 0 to 14. Based on the total sum scores of the scale, level of social support was categorized into three levels (poor = “3–8”, moderate = “9–11”, strong = “12–14”) [21].

In the fourth section, Self Reporting Questionnaire (SRQ-20) was used to measure the level of mental distress [22]. SRQ-20 is a widely used instrument for epidemiological studies in clinical and community settings in Ethiopia and other African countries. The tool is validated in Ethiopian context among perinatal women with good accuracy showing a sensitivity and specificity of 0.86 and 0.76, respectively [23]. It has 20 “Yes” or “No” questions to be used both as self administered and interviewer administered. Its items were scored as “0” (no) and “1” (yes), and the sum score ranges 0–20. Accordingly, pregnant women were considered as screened positive for mental distress if the total sum score of SRQ-20 was 7 or above [24].

The WHO structured questionnaire of Alcohol Use Disorder Identification Test (AUDIT) was the fifth part of the questionnaire used to assess the level of alcohol consumption. AUDIT is a screening tool used to asses alcohol consumption with levels of “no alcohol use”, “social use”, “harmful drinking”, “hazardous drinking” and “alcohol dependency” with ascending order of alcohol use severity [25]. AUDIT is a cross culturally validated instrument which can be used in different countries with sensitivity and specificity of 0.90 and 0.80, respectively [26, 27]. In the current study, AUDIT had good accuracy and internal consistency as evidenced by a Cronbach alpha = 0.96.

The final section of the questionnaire had questions used to assess the outcome variable (Khat use during current pregnancy). Khat use was assessed based on questions developed from different literatures [28–30]. These questions were supposed to address the duration and the numbers of days pregnant women were chewing Khat per week during their current pregnancy. Individuals with current Khat use history were also asked follow-up questions to mention possible reasons that motivate them to initiate Khat use during their current pregnancy, and they identified more than a single reason. Finally, they were asked about the single most important reason why they initiate to chew Khat currently.

### Data collection procedures

Five data collectors (clinical nurses) and two supervisors (MSc level public health professionals) were participated in the data collection after attending two days of training regarding the contents of the questionnaire and data collection procedures. Prior to the data collection, written consent was obtained from each participant after providing a brief explanation on the scope and objects of the study. Respondents were also informed as they have the right to refuse or withdraw their participation at any time they want, and no any harm could be imposed towards them due to the withdrawal. Data collectors first evaluated the eligibility of each respondent to participate in the study, and only eligible individuals were invited to be interviewed. Pregnant women whose age was equal or greater than 18 years were included where as women with serious illness and difficulty of communication were excluded from the study. During the data collection, personal identifiers like name and phone numbers of respondents never had been recorded. The collected data was also kept confidential and used only for the purpose of the study.

### Data analysis

The completeness and consistency of the collected data was checked first. The data then, entered to Epi-info version 3.5 (software) and exported to a Statistical Package for Social Science (SPSS-version-20) for analysis. We used descriptive statistics to explain study participants in relation to different characteristics and to measure the outcome variable (magnitude of Khat use). These descriptive results were presented using texts, tables and figures. To identify factors associated with Khat use during current pregnancy, bivariable and multi variable analysis were computed. Variables with *P*-values of less than 0.25 in bivariable regression were considered as candidates for multivariable analysis and entered together to the final model. In the multivariable analysis, variables with *P*-values of less than 0.05 were considered as statistically significant correlates of Khat use and the strength of the association was measured by adjusted odds ratio (AOR) with corresponding 95% CI.

## Results

### Socio-demographic characteristics

A total of 718 pregnant women attending antenatal care service participated in the study. The mean (±SD) age of respondents was 27.1(±4.23) years and about 53.5% were within 25–29 years of age range. About 77.6% and 57.5% of pregnant women were married and protestant religion followers, respectively (Table 1).

**Table 1:** Socio-demographic characteristics of pregnant women attending antenatal care services in Gedeo zone health centers, Southern Ethiopia, 2017 (*n* = 718)

Variables	Categories	Frequency	Percentage
Age in years	18–20	42	5.8
	21–24	155	21.6
	25–29	384	53.5
	30–45	137	19.1
Marital status	Married	557	77.6
	Single	161	22.4
Religion	Protestant	413	57.5
	Orthodox	201	28.0
	Muslim	73	10.2
	^a^Others	31	4.3
Ethnicity	Gedeo	412	57.4
	Wolaita	93	13.0
	Gurage	83	11.6
	Oromo	86	12.0
	^b^Others	44	6.1
Residency	Urban	326	45.4
	Rural	392	54.6
Occupation	Farmers	241	33.6
	Employed	166	23.1
	Merchant	121	16.9
	House wife	122	17.0
	^c^Others	68	9.5
Educational status	Unable to read and write	218	30.4
	Able to read and write	171	23.8
	Primary school	125	17.4
	Secondary school	109	15.2
	College and above	95	13.2
Average monthly income	≤ 500	147	20.5
	501–999	159	22.1
	1000–1999	332	46.2
	≥ 2000	80	11.1

^a^ catholic and wakifeta, ^b^ Amhara and Sidama, ^c^ daily laborer and students

### Magnitude of Khat use

The lifetime and current prevalence of khat use among pregnant women attending ANC service were11.0% (95%CI: 8.8–13.2) and 9.9% (95%CI: 7.7–12), respectively. Among current Khat users, 53.5% reported that they chew khat for 2–3 times per week whereas 19.7%, 15.5%, and 11.3% often chew Khat daily, weekly and occasionally, respectively. Demographically, 13.1% pregnant women were within the second trimester pregnancy period. About 12.0% of women having unplanned pregnancy were chewing Khat during their current pregnancy. Similarly, 20.7% of participants whom their partners were chewing Khat and 29.3% of women with alcohol use disorder were khat users.

### Reasons of Khat chewing

**Fig. 1:**
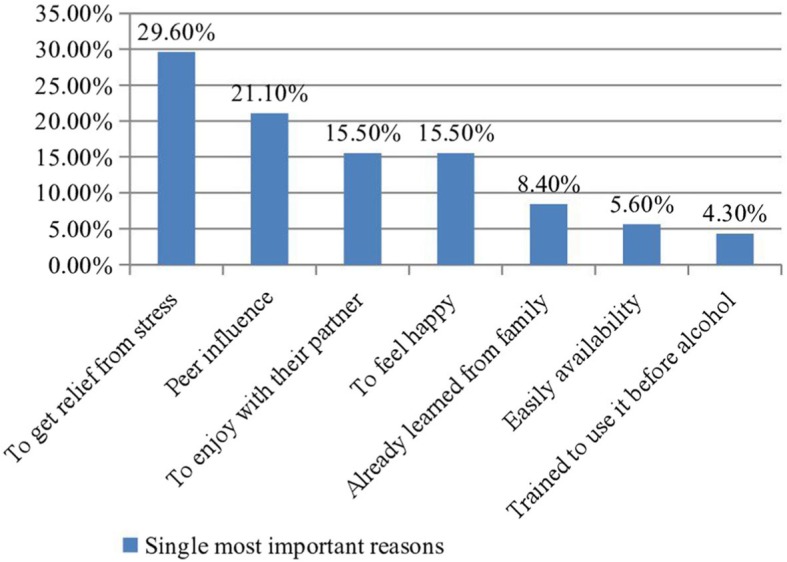
Single most important reason for the initiation of khat use during pregnancy among women attending antenatal care in Gedeo zone rural health centers, Southern Ethiopia (*n* = 71)

There were numerous factors mentioned as reasons of Khat use among pregnant women. Almost all agreed that there is more than one possible reason for the initiation of their chewing practice. About 29.6% and 21.1% of participants considered “getting relief from their psychological stress” and “peer influence” as single most important reasons for chewing, respectively (Fig. 1).

### Factors associated with current Khat use

The outcome of multivariable analysis showed that partner khat use [AOR = 3.450, 95% C.I (1.907–6.244)], alcohol use [AOR = 3.235, 95% C.I (1.573–6.659)] and mental distress [AOR = 3.575, 95% C.I (2.067–6.189)] were factors significantly associated with current Khat use (Table 2).

**Table 2:** Bivariable and multivariable analysis of variables associated with current Khat use among women attending antenatal care in Gedeo zone rural health centers, Southern Ethiopia, 2017

Variables	Categories	Current Khat use status		COR(95% C.I)	AOR(95% CI)	*p*-value
		Yes	No			
Monthly income	≤ 500	17	130	1.00	0.73 (0.33–1.64)	1.00
	501–999	16	143	0.85 (0.42–1.76)	1.10 (0.56–2.15)	0.455
	1000–1999	35	297	0.90 (0.48–1.66)	0.26 (0.07–1.02)	0.779
	≥2000	3	77	0.2 (0.08–1.05)		0.054
Gestational age	First trimester	9	112	0.83 (0.38–1.79)	1.10 (0.47–2.57)	0.814
	Second trimester	29	193	1.55 (0.91–2.64)	1.58 (0.88–2.86)	0.124
	Third trimester	33	342	1.00	1.00	1.00
Pregnancy plan	Planned	39	442	1.00	1.00	1.00
	Unplanned	32	235	1.43 (0.87–2.35)	1.71 (0.97–3.01)	0.060
Partner khat use	Yes	53	289	3.64 (2.09–6.36)	3.45 (1.90–6.24)	0.001*
	No	18	358	1.00	1.00	1.00
Alcohol use	No	59	601	1.00	1.00	1.00
	Yes	12	46	4.65 (2.47–8.73)	3.23 (1.57–6.65)	0.001*
Mental distress	No	32	498	1.00	1.00	1.00
	Yes	39	149	4.07 (2.46–6.73)	3.57 (2.06–6.18)	0.001*
Social support	Poor	25	207	1.55 (0.82–2.92)	1.91 (0.95–3.82)	0.066
	Moderate	28	209	1.71 (0.92–3.19)	1.83 (0.93–3.61)	0.079
	Strong	18	231	1.00	1.00	1.00

* = statistically significant associated variables at ***p*** **< 0.005, 1.00 = Reference**

## Discussion

In this study, the lifetime and current prevalence of Khat use were 11.0% (95%CI: 8.8–13.2) and 9.9% (95% CI: 7.7–12), respectively. The findings of the current study showed a lower prevalence of current Khat use as compared to results of other studies done in Ethiopia among the general population of Jimma town (37.8%) [28] and Butajira [29]. The possible reason for lower prevalence of current Khat use in this study might be due to the effects of health education programs of Dilla University through its Community Based Education (CBE) and Team Training Program (TTP) in which all graduating year medical and health science students are practicing each year. Moreover, Khat use has social desirability concern, especially for females and the higher prevalence in the general population might be due to the higher involvement of adult males in Khat use.

The prevalence of current khat use in this study was also lower than another similar study done in Yemen 40.7% [19]. The possible explanation for this discrepancy of result reports could be due to the socio-cultural difference between study participants. There are different social taboos and cultural restrictions towards female Khat chewers than males and chewing for female were considered as a manner of social deviation in Ethiopia [28]. This might also contribute for the lowered prevalence of Khat use in the current study [28, 29].

The other objective of this study was to identify correlates of Khat use among pregnant women attending ANC service during their current pregnancy. In this regard, having khat user partner, alcohol use behavior and metal distress were found to have significantly significant association with Khat use of pregnant women during their current pregnancy. The odds of using Khat during their current pregnancy were 3.4 times increased among pregnant women whose partners were chewing khat as compared to pregnant women whose partners were not using Khat. This finding is supported by another study conducted in Jimma [30]. The possible explanation for this significant association might be due to the fact that people commonly chew khat within groups gathered together and used as a means of socialization [8, 31]. Hence, individuals may attend chewing ceremonies mainly for socialization at the time they face psychosocial distress and feel loneliness, while not much interested in the chewing. In addition, women may chew khat as a reason for searching friends and to establish close relationships with their spouse by chewing together as a defense mechanism to overcome the pregnancy related stresses [10, 30].

Alcohol users had 3.2 times increased odds of using Khat during pregnancy as compared to their counterparts. This finding is parallel with another study as it can be evidenced by the fact that there is often a common phenomena that people combine other substance use once they have started a single types of psycho active substance [29]. Appropriate education and counseling service is needed regarding the negative effects of alcohol drinking during pregnancy both for the mother and the fetus. Literatures also stated that there is no even minimal amount of alcohol allowed to be consumed during pregnancy [32].

Similarly, women who had mental distress were at 3.6 times odds of using khat during pregnancy as compared to women who had no mental distress. This is consistent with other studies done in Ethiopia [28, 33]. People may prefer Khat use so as to enjoy with the euphoric and psycho stimulant effects of Khat that may help them escape from their distress, sadness, hopelessness which are hallmark symptoms of mental distress [34]. This can have a complicated health outcome for both the mother and the fetus. Thus, professionals are recommended to consider and integrate the prevention, early identification and treatment of mental distress for pregnant women during their antenatal visit [8, 17, 34].

### Strength and limitations of the study

The current study used standardized and validated tools as measurements of variables. Additionally, we used a probability sampling technique and large sample size to make the findings of this study more representative. However, there may be social desirability bias as there are cultural restrictions and social taboos towards female Khat chewers in Ethiopia, especially pregnant woman. This might enforce them to under-report their chewing practice. The cross-sectional nature of the study design might not show the cause and effect relationships between study variables. Therefore, further follow-up study is strongly recommended.

## Conclusions

Significant proportions of participants used Khat during their current pregnancy. Having khat user partner, alcohol use disorder and screened positive for mental distress were significantly associated with Khat use during pregnancy. This demonstrates a need to integrate the prevention, early identification and intervention of Khat use as a component of treatment modality for pregnant women during their antenatal visit.

## Supplementary information

**Additional file 1.** Interview guide used for the data collection.

## Data Availability

The raw data included in the manuscript is available and can be accessed by contacting the corresponding author.

## Abbreviations

ANC: Antenatal Care
AOR: Adjusted odds ratio
AUDIT: Alcohol Use Disorder Identification Test
CI: Confidence interval
COR: Crude odds ratio
DURH: Dilla University Referral Hospital
ETB: Ethiopian Birr
SD: Standard deviation
SRQ: Self Reporting Questionnaire
USA: United State of America
WHO: World Health Organization
