# Supplementary material for: Magnitude of Khat use and associated factors among women attending antenatal care in Gedeo zone health centers, southern Ethiopia: a facility based cross sectional study
Source: BMC Public Health. 2020 Jan 28;20:110. doi: 10.1186/s12889-019-8026-0 (PMC6988234; doi:10.1186/s12889-019-8026-0)
Supplement: Supplementary file 1 — Additional file 1. Interview guide used for the data collection. [file 12889_2019_8026_MOESM1_ESM.docx]

Interview guide used for the data collection of the study

Part-I- demographic and Socio-economic Characteristics

| No | Questions | Answers |
| --- | --- | --- |
| 101 | Age |  |
| 102 | Marital status | 1. Married 2.Single |
| 103 | Religion | 1.Orthodox 2.Muslim  3.Protestant 4.Catholic  5. Others* |
| 104 | Ethnicity | 1.Gedeo 2.Oromo  3. Amhara 4.Gurage 5.Tigre  5.sidama 6.wolaita 7.Others* |
| 105 | Occupational status(level) | 1. Housewife 2.Farmer  3. Employer 4. Student  5. Daily labourer 6. Merchant  7. Jobless 8. other (specify) |
| 106 | Educational level | 1 not able to write and read  2 able to write and read but not engage in regular school  3 Primary school  4 Secondary school  5 Higher educations |
| 107 | Monthly income | …………….birr |
| 108 | Residency | - 1. Urban 2. Rural |

Part-II- obstetric related factors

| No. | Questions | Answers |
| --- | --- | --- |
| 201 | No of Parity (whether- aborted, still birth, normal) | ------------------ |
| 202 | History of abortion? | 1. Yes 2. No |
| 203 | No of children? | ----------------- |
| 204 | Have you faced History of still birth? | 1. Yes 2. No |
| 205 | Gestational age? | ----------------- |
| 206 | Is your current pregnancy planed? | 1. Yes 2. No |
| 207 | Is there partner use of khat? | 1. Yes 2. No |

Part-III- the 3-Item Oslo Social Support Scale (OSS-3)

| 301 | How many people are so close to you that you can count on them if you have serious problem?(select only one) | 1. none |
| --- | --- | --- |
|  |  | 2. one or two |
|  |  | 3. 3-5 |
|  |  | 4. above 5 |
| 302 | How much concern do people show in what you are doing?(select only one) | 5.a lot |
|  |  | 4. some |
|  |  | 3. uncertain |
|  |  | 2. little |
|  |  | 1.none |
| 303 | How easy can you get help from neighbours if you should need it?(select only one) | 5. very easy |
|  |  | 4. easy |
|  |  | 3. possible |
|  |  | 2. difficult |
|  |  | 1.very difficult |

Part- IV. Mental distress questionnaires

| **No** | **Encountered health problem within the last 4 weeks** | **Yes** | **No** |
| --- | --- | --- | --- |
| 401 | Do you often have headache? | 1 | 0 |
| 402 | Is your appetite poor? | 1 | 0 |
| 403 | Do you sleep badly? | 1 | 0 |
| 404 | Are you easily frightened? | 1 | 0 |
| 405 | Do your hands shake? | 1 | 0 |
| 406 | Do you feel nervous, tens or worried? | 1 | 0 |
| 407 | Is your digestion poor? | 1 | 0 |
| 408 | Do you have trouble thinking clearly? | 1 | 0 |
| 409 | Do you unhappy? | 1 | 0 |
| 410 | Do you cry more than usual? | 1 | 0 |
| 411 | Do you find it difficult to enjoy your daily activities? | 1 | 0 |
| 412 | Do you find difficult in decision making in day to day life? | 1 | 0 |
| 413 | Is your daily work suffering? | 1 | 0 |
| 414 | Are you unable to play useful part in life? | 1 | 0 |
| 415 | Have you lost interest in things? | 1 | 0 |
| 416 | Do you feel that you are a worthless person? | 1 | 0 |
| 417 | Has the thought of ending your life been on your mind? | 1 | 0 |
| 418 | Do you feel tired all the time? | 1 | 0 |
| 419 | Do you have uncomfortable feelings in your stomach? | 1 | 0 |
| 420 | Are you easily tired? | 1 | 0 |
|  | Total numbers of “yes” |  |  |

Part-V- Alcohol use disorder identification test questionnaires.

| No. | Questions | Answers |
| --- | --- | --- |
| 501 | How often do you have a drink containing alcohol? | (0)Never  (1) Monthly  (2) 2–4 times a month  (3) 2–3 times a week  (4) 4 or more times a week |
| 502 | How many drinks containing alcohol do you have on a typical day when you are drinking? | (0)1–2  (1) 3 or 4  (2) 5 or 6  (3) 7–9  (4) 10 or more |
| 503 | How often do you have six or more drinks on one occasion? | (0)never  (1) less than monthly  (2) monthly  (3) weekly  (4) daily or almost daily |
| 504 | How often during the last year have you found that you were unable to stop drinking once you started? | (0)never  (1) less than monthly  (2) monthly  (3) weekly  (4) daily or almost daily |
| 505 | How often during the last year have you failed to do what was normally expected of you because of drinking? | (0) never  (1) less than monthly  (2) monthly  (3) weekly  (4) daily or almost daily |
| 506 | How often during the last year have you needed a first drink in the morning to get yourself going after a heavy drinking session? | (0)never  (1) less than monthly  (2) monthly  (3) weekly  (4) daily or almost daily |
| 507 | How often during the last year have you felt guilt or remorse after drinking? | (0)never  (1) less than monthly  (2) monthly  (3) weekly  (4) daily or almost daily |
| 508 | How often during the last year have you been unable to remember what happened the night before because of drinking? | (0) never  (1) less than monthly  (2) monthly  (3) weekly  (4) daily or almost daily |
| 509 | Have you or someone else been injured as the result of your drinking? | (0)no  (2) yes, but not in the last year  (4) yes, during the last year |
| 510 | Has a friend, relative, or doctor or other health worker been concerned about your drinking or suggested you cut down? | (0) no  (2) yes, but not in the last year  (4) yes, during the last year |

Part- VI- Khat chewing related questions

| 601 | Have you ever chewed khat? | 1. Yes 2. No |  |
| --- | --- | --- | --- |
| 602 | Have you chewed khat during your current pregnancy? | 1. Yes 2. No |  |
| 603 | If yes for Q number 102, the frequency of your chewing? | 1. Every day 2. 2-3 times a week 3. One times a week 4. Others --------- |  |
| 604 | Amount of khat in local measurement (zurba) or cost of khat chewed at one time? |  |  |
| 605 | Time spent to chew khat for one episode? |  |  |
| 606 | Reason to start khat chewing? |  |  |
| 607 | What is the single most important reason to initiate khat chewing? |  |  |
